# Digitalizing the Microbiome for Human Health

**DOI:** 10.1128/mSystems.00129-19

**Published:** 2019-06-04

**Authors:** Kirti Nath, Christoph A. Thaiss

**Affiliations:** a Microbiology Department, Perelman School of Medicine, University of Pennsylvania, Philadelphia, Pennsylvania, USA

**Keywords:** MWAS, artificial intelligence, disease, health, microbiome

## Abstract

The microbiome has recently joined the club of endocrine entities of the human body that are involved in homeostasis and disease. Microbiome characterizations are now typically included in longitudinal and cross-sectional population studies, associations with microbiome features have been made for almost any human disease, and the molecules by which the microbiome functionally contributes to host physiology are being elucidated. The leverage of these efforts for human health, however, is still rather modest. In this Perspective, we summarize some of the challenges that need to be overcome in order to make microbiome studies as informative for human health as genetic studies. Focusing on the role of the microbiome in host metabolism and inflammation, we also outline potential strategies that can be employed to achieve the next milestones in the journey toward microbiome-informed human health assessment and action.

## Abstract (precis)

The microbiome has recently joined the club of endocrine entities of the human body that are involved in homeostasis and disease. Microbiome characterizations are now typically included in longitudinal and cross-sectional population studies, associations with microbiome features have been made for almost any human disease, and the molecules by which the microbiome functionally contributes to host physiology are being elucidated.

## PERSPECTIVE

The successful completion of the human genome project over 15 years ago represented a milestone in medicine and enabled human diseases to be traced to causal genetic variants, allowing treatments to be developed that were more specific and more targeted. This has led to a wealth of genome-wide association studies (GWAS) which enabled the characterization of disease-associated variances in the human genome. GWAS, in turn, facilitated the study of disease genes and their role in the complex pathogeneses of human diseases. However, two decades of GWAS have also fortified the notion that the most prevalent human disorders, such as cardiovascular disease, type II diabetes, obesity, neurodegeneration, and chronic inflammatory diseases, cannot be fully explained by genetic variation (1). Rather, these diseases are not only polygenic but also strongly influenced by environmental factors, as exemplified by monozygotic twins discordant for their individual disease susceptibilities. In contrast to the contributions that genetic variances make to human phenotypes, the specific mechanisms and molecules by which environmental exposure and lifestyle modulate disease susceptibility have remained unclear.

**FIG 1:**
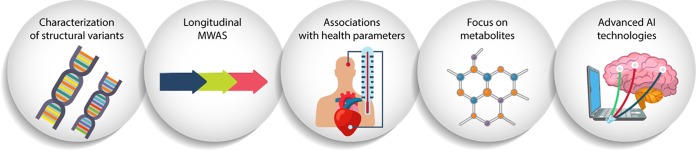
Schematic of five areas of microbiome research undergoing active developments toward harnessing the metagenomic influence on host health.

The realization that the microbiome connects the outside world and the body’s physiology in multiple ways—by digesting food, metabolizing xenobiotics, and providing colonization resistance, among many other functions—has opened the door for a more detailed mechanistic understanding of how the environment signals to the body. This understanding has provided the ground for the identification of distinct microbial species or microbial molecules that can subsequently be targeted for therapeutic purposes, analogous to traditional modulations of aberrantly abundant or dysfunctional host-derived molecules in various disease contexts. Indeed, microbiome-wide association studies (MWAS) have attempted to pursue a GWAS-like strategy in order to find associations between the metagenome and phenotypic traits (2). Numerous examples have since been found in which specific taxonomic features of the microbiota statistically associated with a particular health outcome. Why then have MWAS not yet been translated into clinical applications and applied pharmacology? Inflammatory bowel diseases (IBD) may serve as a good example, given the clear role of the microbiome in its pathophysiology. Soon after completion of the human genome project, IBD susceptibility genes were discovered, among them, NOD2 (3), the interleukin-23R (IL-23R) pathway (4), and the autophagy gene ATG16L1 (5, 6). These have since served as actionable drug targets in clinical trials. While MWAS of IBD did yield significant associations of microbial taxa with disease outcome (7), it has been less obvious how to harness this knowledge for combating the disease. While the sheer time that it takes from basic discovery to clinical application is certainly one of the reasons why recent MWAS have not yet manifested in palpable clinical outcomes, there are multiple additional reasons why MWAS have so far led to fewer actionable insights than GWAS. In the following, we highlight some of the field’s challenges for the coming decade and outline strategies by which they might be overcome (Fig. 1).

First, the human genome is represented by a linear string of characters with “digital” variances. Associating alterations in this linear sequence with phenotypic traits is conceptually and computationally intuitive. In the case of the microbiome, the situation is far more “analog” and complex, since numerous parallel and partially overlapping linear genomes constitute the metagenome. The relative abundances and phenotypic contributions of these individual genomes are highly variable and idiosyncratic. Rather than employing nucleotide variances, MWAS have therefore primarily relied on linking relative abundances of genes (and, in many cases, only taxonomic groups) with disease phenotypes, which is less informative in terms of protein function than polymorphisms in a linear genome. “Digitalizing” the microbiome to enhance the resolution of MWAS can be achieved in multiple ways: linking phenotypic traits to microbial copy number variations, focusing on strain-level variances in bacterial genomes, tracing horizontal gene transfers through long-read sequencing, and advancing our understanding of genome-to-proteome relationships in the microbiome. Recent explorations of structural variants in the microbiome have indicated that such an approach provides an important layer of information that is associated with host health (8). Routinely incorporating these into MWAS will greatly enhance their power and sensitivity, thereby getting closer to the conceptual analog of a “metagenomic single-nucleotide polymorphism.”

Second, in contrast to the host genome, which remains constant over the organism’s life span (perhaps with the exception of accumulated somatic mutations), the microbiome is highly dynamic in both the temporal and spatial dimension. Several body sites are colonized by commensal microorganisms, including the skin and the gastrointestinal, respiratory, and urogenital tracts. The relative contributions of the metagenomes at all of these sites to human health are not easily quantified. Even within each of these organs, the microbiome displays strong regional features and is unequally distributed with respect to both taxonomic composition and functional output. Furthermore, the microbial communities are evolving over time, with a profound impact on host health (9). As such, cross-sectional association studies in analogy to GWAS are less likely to achieve an accurate representation of the microbial genetic predisposition to a disease. Rather, longitudinally performed MWAS might enable us to identify intermediate time points associated with the initiation of a disease state. These transient microbiome configurations might be more informative than stable states which might be a consequence rather than cause of a disease (10).

Third, the majority of MWAS have focused on clinically defined endpoints, such as liver cirrhosis, atherosclerosis, or Crohn’s disease, among many others (2). While using such clinically defined endpoints is plausible for GWAS, where the underlying assumption is that a genetic variance directly correlates with disease risk in a digital manner, this conjecture is less applicable to the metagenome. Microbiome variations often influence host biological processes in subtler, more finely grained ways than genetic alterations. As such, microbiome-controlled variances in host physiology and pathophysiology may not always fall into the categories associated with clinically diagnosed diseases. As a result, associations of clinical endpoints with specific metagenomic features are harder to achieve, especially in light of the temporal and spatial dynamics discussed above. For instance, if the function of an enzyme is essential in the pathophysiology of a disease and if a genetic variant leads to loss of function of the enzyme-encoding gene, then a GWAS approach will detect the variant as disease associated. In contrast, microbiome-derived molecules may modulate the function of this enzyme in ways that are more subtle than clear gain or loss of function. Attempting to reduce the effects of the microbiome to clinically defined endpoints introduces analytical tunnel vision in categorizing metagenomic effects. Moving forward, the field will greatly benefit from shifting the focus from disease phenotypes to broader health parameters. For instance, instead of determining the microbiome impact on atherosclerosis, it might be more informative to associate microbiome features with blood cholesterol levels, vascular macrophage biology, systolic blood pressure, etc. Indeed, the initial MWAS of individual parameters of host physiology have shown great promise (11) and will be a useful tool to disentangle specific metagenomic influences from composite disease outcomes. This more extensive focus on metagenome associations with health rather than with disease will be facilitated by inclusion of microbiome features in electronic health records. Similar to personal lifestyle elements such as smoking, diet, and physical activity, recording microbiome features associated with health parameters across the human population will prove immensely informative with regard to determining metagenomic states that are optimal for human health.

Fourth, given the difficulty in stably modulating the microbiota for therapeutic purposes, focusing on microbiota-derived metabolites in MWAS is emerging as a powerful strategy to link disease outcomes to the functional (rather than taxonomic) state of the microbiome (12). Indeed, this approach has started to yield functional insights into disease etiologies (13) that not only go beyond knowledge about the microbial ecosystem achieved by taxonomic survey but also provide a more direct view of potential therapeutic interventions.

Finally, the use of artificial intelligence (AI) approaches has enabled microbiome-based predictions of phenotypic outcomes (14). These are valuable in assessing those microbiome variables with the highest contribution to predictive power. However, several challenges remain, including those represented by cases in which (i) the structured data are more limited in availability, (b) the outcome is highly dimensional and phenotypically complex, and (c) the outcome is nonstatic in nature, i.e., changes are desirable. The latter point is essential for the ability of AI technologies not only to map influences of the microbiome on human health parameters but also to harness this knowledge and provide actionable insights of clinical relevance. Areas of computational development, including deep neural networks, along with the generation of large databases of information regarding host-microbiome interactions may facilitate this development (15). New machine learning strategies, such as those using ecological principles to infer the likelihood for a particular microbe to impact intestinal microbial ecology, together with information about within-host microbiome evolution and microbiome adaptation to different environmental conditions, may not only determine significant microbiome parameters which influence host physiology but also predict the effects of modulating these parameters in individuals.

Together, these steps toward digitalization of the microbiome will greatly improve our ability to derive clinically and pharmacologically meaningful action items from microbiome surveys. Microbiome science has introduced numerous revolutionary concepts of how we think about many aspects of human physiology. The time is ripe to use these insights to start revolutionizing many aspects of human medicine.

## References

[1] Walley AJ, Asher JE, Froguel P 2009 The genetic contribution to non-syndromic human obesity. Nat Rev Genet 10:431–442. doi:10.1038/nrg2594. 19506576

[2] Gilbert JA, Quinn RA, Debelius J, Xu ZZ, Morton J, Garg N, Jansson JK, Dorrestein PC, Knight R 2016 Microbiome-wide association studies link dynamic microbial consortia to disease. Nature 535:94–103. doi:10.1038/nature18850. 27383984

[3] Ogura Y, Bonen DK, Inohara N, Nicolae DL, Chen FF, Ramos R, Britton H, Moran T, Karaliuskas R, Duerr RH, Achkar JP, Brant SR, Bayless TM, Kirschner BS, Hanauer SB, Nunez G, Cho JH 2001 A frameshift mutation in NOD2 associated with susceptibility to Crohn's disease. Nature 411:603–606. doi:10.1038/35079114. 11385577

[4] Duerr RH, Taylor KD, Brant SR, Rioux JD, Silverberg MS, Daly MJ, Steinhart AH, Abraham C, Regueiro M, Griffiths A, Dassopoulos T, Bitton A, Yang H, Targan S, Datta LW, Kistner EO, Schumm LP, Lee AT, Gregersen PK, Barmada MM, Rotter JI, Nicolae DL, Cho JH 2006 A genome-wide association study identifies IL23R as an inflammatory bowel disease gene. Science 314:1461–1463. doi:10.1126/science.1135245. 17068223 PMC4410764

[5] Hampe J, Franke A, Rosenstiel P, Till A, Teuber M, Huse K, Albrecht M, Mayr G, De La Vega FM, Briggs J, Gunther S, Prescott NJ, Onnie CM, Hasler R, Sipos B, Folsch UR, Lengauer T, Platzer M, Mathew CG, Krawczak M, Schreiber S 2007 A genome-wide association scan of nonsynonymous SNPs identifies a susceptibility variant for Crohn disease in ATG16L1. Nat Genet 39:207–211. doi:10.1038/ng1954. 17200669

[6] Rioux JD, Xavier RJ, Taylor KD, Silverberg MS, Goyette P, Huett A, Green T, Kuballa P, Barmada MM, Datta LW, Shugart YY, Griffiths AM, Targan SR, Ippoliti AF, Bernard EJ, Mei L, Nicolae DL, Regueiro M, Schumm LP, Steinhart AH, Rotter JI, Duerr RH, Cho JH, Daly MJ, Brant SR 2007 Genome-wide association study identifies new susceptibility loci for Crohn disease and implicates autophagy in disease pathogenesis. Nat Genet 39:596–604. doi:10.1038/ng2032. 17435756 PMC2757939

[7] Gevers D, Kugathasan S, Denson LA, Vazquez-Baeza Y, Van Treuren W, Ren B, Schwager E, Knights D, Song SJ, Yassour M, Morgan XC, Kostic AD, Luo C, Gonzalez A, McDonald D, Haberman Y, Walters T, Baker S, Rosh J, Stephens M, Heyman M, Markowitz J, Baldassano R, Griffiths A, Sylvester F, Mack D, Kim S, Crandall W, Hyams J, Huttenhower C, Knight R, Xavier RJ 2014 The treatment-naive microbiome in new-onset Crohn's disease. Cell Host Microbe 15:382–392. doi:10.1016/j.chom.2014.02.005. 24629344 PMC4059512

[8] Zeevi D, Korem T, Godneva A, Bar N, Kurilshikov A, Lotan-Pompan M, Weinberger A, Fu J, Wijmenga C, Zhernakova A, Segal E 2019 Structural variation in the gut microbiome associates with host health. Nature 568:43–48. doi:10.1038/s41586-019-1065-y. 30918406

[9] Thaiss CA 2018 Microbiome dynamics in obesity. Science 362:903–904. doi:10.1126/science.aav6870. 30467161

[10] Uhr GT, Dohnalova L, Thaiss CA 2019 The dimension of time in host-microbiome interactions. mSystems 4:e00216-18. doi:10.1128/mSystems.00216-18. PMC6381226 30801030

[11] Rothschild D, Weissbrod O, Barkan E, Kurilshikov A, Korem T, Zeevi D, Costea PI, Godneva A, Kalka IN, Bar N, Shilo S, Lador D, Vila AV, Zmora N, Pevsner-Fischer M, Israeli D, Kosower N, Malka G, Wolf BC, Avnit-Sagi T, Lotan-Pompan M, Weinberger A, Halpern Z, Carmi S, Fu J, Wijmenga C, Zhernakova A, Elinav E, Segal E 2018 Environment dominates over host genetics in shaping human gut microbiota. Nature 555:210–215. doi:10.1038/nature25973. 29489753

[12] Thaiss CA, Elinav E 2017 The remedy within: will the microbiome fulfill its therapeutic promise? J Mol Med (Berl) 95:1021–1027. doi:10.1007/s00109-017-1563-z. 28656322

[13] Franzosa EA, Sirota-Madi A, Avila-Pacheco J, Fornelos N, Haiser HJ, Reinker S, Vatanen T, Hall AB, Mallick H, McIver LJ, Sauk JS, Wilson RG, Stevens BW, Scott JM, Pierce K, Deik AA, Bullock K, Imhann F, Porter JA, Zhernakova A, Fu J, Weersma RK, Wijmenga C, Clish CB, Vlamakis H, Huttenhower C, Xavier RJ 2019 Gut microbiome structure and metabolic activity in inflammatory bowel disease. Nat Microbiol 4:293–305. doi:10.1038/s41564-018-0306-4. 30531976 PMC6342642

[14] Thaiss CA, Itav S, Rothschild D, Meijer M, Levy M, Moresi C, Dohnalova L, Braverman S, Rozin S, Malitsky S, Dori-Bachash M, Kuperman Y, Biton I, Gertler A, Harmelin A, Shapiro H, Halpern Z, Aharoni A, Segal E, Elinav E 2016 Persistent microbiome alterations modulate the rate of post-dieting weight regain. Nature 540:544–551. doi:10.1038/nature20796. 27906159

[15] Eraslan G, Avsec Z, Gagneur J, Theis FJ 2019 Deep learning: new computational modelling techniques for genomics. Nat Rev Genet doi:10.1038/s41576-019-0122-6. 30971806

